# Psychosocial interventions for suicidal ideation, plans, and attempts: A database of randomised controlled trials

**DOI:** 10.1186/1471-244X-14-86

**Published:** 2014-03-25

**Authors:** Helen Christensen, Alison L Calear, Bregje Van Spijker, John Gosling, Katherine Petrie, Tara Donker, Katherine Fenton

**Affiliations:** 1 Black Dog Institute, University of New South Wales, Hospital Road, Randwick, Sydney, New South Wales 2031, Australia; 2 Centre for Mental Health Research, The Australian National University, Canberra, Australia; 3 Department of Clinical Psychology, VU University Amsterdam, Amsterdam, The Netherlands; 4 EMGO Institute for Health and Care Research, VU University Amsterdam and VU University Medical Center, Amsterdam, The Netherlands

**Keywords:** Suicide, Psychological interventions, Randomised controlled trials, Prevention, Database, Meta-analysis, Systematic review

## Abstract

**Background:**

Research in suicide prevention using psychosocial interventions is rapidly advancing. However, randomised controlled trials are published across a range of medical, psychological and sociology journals, and it can be difficult to locate a full set of research studies. In this paper, we present a database of randomised controlled outcome studies on psychosocial interventions targeting suicidal behaviour. The database is updated annually and can be accessed by contacting the corresponding author.

**Description:**

A comprehensive literature search of the major bibliographical databases (PsycINFO; PubMed; Cochrane Central Register of Controlled Trials) was conducted for articles published between 1800 to July 30 2013, and examined reference lists of previous relevant reviews and included papers to locate additional references. Studies were included if they featured a randomised controlled design in which the effects of a psychosocial intervention were compared to a control condition (no intervention, attention placebo, wait-list, treatment-as-usual [TAU]), another psychosocial intervention or a pharmacological intervention. In total, 12,250 abstracts were identified. Of these, 131 studies met eligibility criteria and were included. Each paper was then coded into categories of participant characteristics (age, gender, formal diagnosis, primary reason for recruitment); details of the intervention (recruitment setting, content, intervention setting, administering individual, delivery type, delivery format, delivery frequency, delivery length); and study characteristics (control and experimental conditions, primary outcome/s, secondary outcome/s, follow-up period). One paper has been published from the database using studies collected and coded prior to 2012.

**Conclusion:**

The database and listing of 131 studies is available for use by suicide prevention researchers. It provides a strong starting point for systematic reviews and meta-analyses of treatments and interventions. It will be updated yearly by researchers funded through the Australian National Health and Medical Research Council Centre for Research Excellence for Suicide Prevention (CRESP), located at the Black Dog Institute, Australia. This database adds to the evidence base of best-practice psychosocial interventions for suicidal behaviour and prevention.

## Background

Suicidal behaviour is a major public health problem worldwide [1] causing significant burden [2,3]. Overall, there is still a considerable lack of understanding of causal mechanisms underlying suicidal thoughts and behaviours and a paucity of effective, rigorously evaluated treatment and prevention strategies for suicide [4-7]. For these reasons, it is vital to consolidate our knowledge base to fill these gaps, better understand causal mechanisms and reduce the global burden of suicide.

Numerous systematic reviews looking at suicide prevention strategies have been conducted to date, often targeting specific populations [8-10], specific treatments [11-13] or employing other restrictions such as country where included studies are conducted. Intervention studies using gold-standard methodology (i.e. randomised controlled designs [RCTs]) are relatively limited in suicide prevention [14,15]. Individual research studies are published across medical, psychological, sociological and generalist journals.

This paper describes the development of a database of Randomised Controlled Trials (RCTs) of psychosocial interventions for suicidal thoughts and behaviour. The database aims to systematically compile and update RCTs from these various sources, and provide a resource to researchers into the future. Similar databases have been established to allow researchers to undertake meta-analyses of anxiety and depression interventions and treatments [16,17].

The database, and all the studies within it, can be accessed freely via request to the corresponding author. We believe the database will facilitate future systematic reviews and meta-analyses into psychosocial interventions for suicidal thoughts and behaviour, as well as stimulate future research in suicide prevention. The current paper outlines the methodology used to construct the database and describes the characteristics of coded studies.

## Construction and content

### Identification and selection of studies

A comprehensive literature search of three databases (Cochrane Central Register of Controlled Trials [CENTRAL], PsycINFO and PubMed) was conducted for articles published in the period 1800 to July 30 2013 using the key word search string ‘Suicid*’ OR ‘self-harm’ OR ‘self-poisoning’ AND ‘Trial’ OR ‘intervention’ OR ‘prevention’. The search was limited to ‘humans’, ‘English’ and ‘peer-reviewed journals’.

To date, four iterations of this search have been conducted and are detailed in Table 1. The original search was conducted in April 2009, and was updated in July 2010, December 2010 and December 2011. A further update was undertaken in July 2013. Exact search terms and the number of abstracts identified in each database are presented in Table 2. Data presented henceforth refers to the overall total of these five searches. In total, 12, 250 abstracts were identified (n = 11, 600 after removal of duplicates).

**Table 1:** Number of abstracts identified at each search

	***Number of abstracts***				
	**Original search**	**First update**	**Second update**	**Third update**	**Fourth update**
	**1800 – April 3 2009**	**April 3 2009 – July 2010**	**July 2010 – December 31 2010**	**January 1 2011 – December 31 2011**	**January 1 2012 – July 30 2013**
***Database***					
CENTRAL		69	39	24	4
PsycInfo		574	424	426	31
PubMed		606	95	278	687
TOTAL	8986	1249	558	728	722

**Table 2:** **Search terms and total number of abstracts identified per source**^**a)**^

***Database***	***Search String***	***Number of abstracts***
**CENTRAL**^**b)**^	Suicid*’ or ‘self-harm’ or ‘self-poisoning’ and ‘Trial’ or ‘intervention’ or ‘prevention’	12,236
**PUBMED**	“	
**PSYCINFO**	“	
**Additional sources (hand searched)**		7
	Total	12,250
	After removal of duplicates	11,600

^a)^All searches were conducted until July 30 2013.

^b)^Cochrane Central Register of Controlled Trials.

### Inclusion of studies

The inclusion criteria for the current review included (a) the program trialled was a psychosocial intervention for the treatment or prevention of suicidal behaviour, (b) suicidal behaviour (self-harm, ideation, attempt or completion) was a primary or secondary outcome measure for the trial, (c) the study was a randomised controlled trial with a no intervention, wait-list, attention or treatment as usual (TAU) control condition, and (d) the trial was published in a peer-reviewed, English language journal. Trials of pharmacological interventions were only included if they also contained a psychosocial component. No restrictions were placed on participant age, mode of recruitment, medication status or psychiatric diagnosis. To be included, studies must have included an outcome measure of suicidal behaviour. Where this was a secondary outcome, the primary outcome must have been a mental health measure.

A psychosocial intervention was defined as an intervention that provided psychotherapy (e.g., Cognitive Behaviour Therapy [CBT], Dialectical Behaviour Therapy [DBT], psychoeducation, supportive counselling, community treatment or case management [including Assertive Community Treatment]). The intervention could be individual or group-based, delivered face-to-face or distally, and in any setting. Studies were excluded if the intervention did not directly target the individual receiving the intervention. As such, gatekeeper programs were not included unless they provided data on the mental health outcomes of at-risk populations (and not improvements in the trained workforce). There are a broad range of issues surrounding the definition and nature of suicidal behaviour. For the purposes of constructing this database, we considered all suicide and related behaviours to be outcome measures, using the terms described by each individual article.

### Screening process

**Figure 1:**
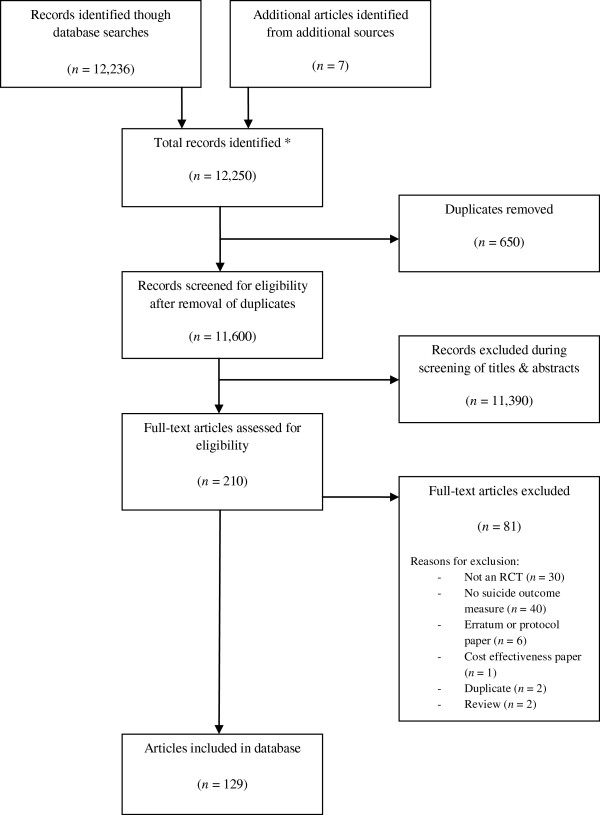
**Stages in identification of articles relevant to database.** *All searches conducted 1800 – June 30 2013.

A total of 12,250 abstracts were identified through the database search. Additionally, reference lists of full text papers, and of previous systematic reviews of psychosocial interventions for suicide, were examined for potential papers. Seven additional references were identified and primary studies collected for further analyses. After removal of duplicates, 11,600 abstract records remained. These 11,600 titles and abstracts were screened for inclusion by two independent reviewers. Of these, 11,390 were deemed ineligible for inclusion, as they did not fulfil one or more of the inclusion criteria. Full text articles were obtained for the remaining 210 abstracts and each screened for inclusion by two independent reviewers. Discrepancies between reviewers were opened to discussion by the research team and resolved by consensus primarily through reference to the research paper. A total of 131 of these studies met all inclusion criteria and were entered into the database. A flowchart detailing this process is provided in Figure 1.

### Data extraction

Data extraction of the included studies was completed by two independent reviewers. Any disagreements were resolved through discussion with reference to the original publication. On the occasion where agreement could not be reached, a third or fourth reviewer was consulted. The following characteristics were recorded for each study.

#### Characteristics of participants

• *Age*: Mean age of participants was recorded if it could be determined. Additionally, the age bracket of participants was noted if available. The closest age bracket was selected according to the following categories (12–25 years: adolescent/young adult, 18–64 years: adult, 65+ years: older adult).

• *Sex:* Participant sex was recorded as the proportion of participants that were male (if not available, NA was reported).

• *Reason for recruitment*: The primary reason for participant recruitment to the trial was noted, in terms of either a psychiatric diagnosis or suicidal behaviour (suicidal ideation or suicide attempt [including hospitalisation for deliberate self-harm]). There was no restriction on the currency of the suicidal behaviour/ideation.

• *Formal diagnosis*: The formal diagnosis of the majority (at least 70%) of the sample was recorded (if relevant). The diagnoses coded were: depression, anxiety, borderline personality disorder, psychosis/schizophrenia, and substance use disorder. It was also noted whether any participants were excluded from the study on the basis of very high suicidal ideation.

#### Characteristics of the intervention

• *Recruitment setting*: The setting in which participants were recruited to the trial was coded. This included clinical referrals (general practice, private/specialist mental health clinics, hospitals), schools and open community recruitment.

• *Intervention setting*: The setting in which the intervention was delivered was recorded. These settings were similar to those described for ‘recruitment setting’ but could be different.

• *Intervention content*: The psychosocial intervention described and evaluated in the study was classified, based on the description provided in the paper. Nine core categories of intervention content were identified and coded. Additional intervention content was categorised as ‘other’ and a description provided. A proportion of the studies described ‘quality improvement’ activities; that is, they evaluated changes to mental health systems or enhanced practices. These studies were coded as ‘quality improvement’ in order to capture this broad category.

• *Control intervention*: The type of control condition employed in the study was coded. This included no-intervention, wait-list, attention and treatment as usual (TAU).

• *Additional intervention*: This category describes any other interventions that were concurrently evaluated in the study, such as an additional psychosocial intervention or a pharmacological intervention.

• *Delivery type*: The manner in which the intervention was delivered (e.g., face-to-face, telephone, postcard, booklet) was recorded.

• *Delivery length*: The number of scheduled face-to-face sessions and/or distal (non face-to-face) sessions was recorded, if available. If a set number of sessions was not provided for in the intervention, then the range of session length was recorded or ‘variable’ was stated.

• *Delivery format*: This coded the format of the treatment, such as individual therapy, group therapy or family therapy.

• *Intervention facilitator:* Professional background of the individual delivering the intervention (e.g., psychologist, teacher, nurse) was coded.

#### Study characteristics

• *Outcomes*: The primary and secondary outcomes measured in the study were coded. These included suicidal behaviour (e.g., suicidal ideation/thoughts, plans of suicide, suicide attempts, suicide completion, self-harm), as well as more general mental health outcomes (e.g., depressive symptoms, hopelessness). All stated primary and secondary outcomes were recorded. Agreement was reached on the primary outcome by the research reviewers, if authors failed to state its nature.

• *Follow-up period*: The time points in the study at which participants completed outcome measures was recorded.

## Discussion and utility

This paper describes the construction and content of an online database of randomised controlled trials of psychosocial interventions targeting suicidal thoughts and/or behaviours. The database is updated annually.

The usefulness of such a database is supported by the high level of research interest in similar databases, such as that of Cuijpers and colleagues’ database in the field of depression intervention [17]. Like Cuijpers et al.’s [16] database, which has been designated ‘highly accessed’ by BMC Psychiatry, the current database has the potential to stimulate an increase in the efficiency, relevance and quality of reviews in the area of suicide prevention, as well as informing future research directions. A series of systematic reviews are currently in preparation by members of the database team based on studies included in the database. To date, one review has been published on suicide prevention in schizophrenic spectrum disorders and psychosis on trials identified in 2011 [18].

Certain limitations are inherent in such a database. Firstly, relevant papers may have been missed due to the search strategy. Secondly, this database does not provide effect sizes or quantitative measures of differences between control and intervention groups. Because different statistical approaches are indicated, depending on the number of studies and the quality of the research trials, these indicators will need to be drawn directly from the research papers by the relevant research groups. Similarly, coded variables included may not be those of interest to particular researchers, and new variables may need to be coded, and current variables recoded. Nevertheless, variables included in this database will be reviewed upon each update, with variables such as study quality and inclusion/calculation of effect sizes to be introduced at later iterations. There is also no guarantee even with two coders that each entry is accurate, when data provided is incomplete or poor quality.

By providing researchers with access to such a database, inadvertent overlap by different research teams conducting similar reviews can be averted, meaning that resources can be better allocated. Access to an up-to-date database will further save valuable time and resources needed to conduct systematic searches, screen abstracts, and code papers for each new review conducted. This database will also allow independent reanalysis of prior findings and therefore increase transparency and accountability of research.

Perhaps more importantly, though, this database will allow for a comprehensive overview of the existing knowledge in the suicide prevention field, providing a synthesis of all published RCTs and highlighting thematic consistencies as well as uncovering ambiguities or inconsistencies. The growing number of studies in this area, which in turn increases the difficulty associated with conducting systematic reviews in the area, and the fact that the few systematic reviews that have been conducted to date have focused on specific interventions or populations, reinforces the need for good records. Flowing from this, more targeted research questions can be addressed through well-informed systematic reviews, enabling comprehensive, up-to-date knowledge of what specifically works, under what circumstances, for whom, and what factors either facilitate or hinder such efforts.

## Conclusion

The present database collates randomised controlled trials of suicide prevention. As such it has the potential to facilitate better informed and thus better designed treatment and prevention strategies for suicidal thoughts and behaviours that serve, more broadly, to reduce the global burden of suicide.

## Competing interests

The authors declare that they have no competing interests.

## Authors’ contributions

*Database*: HC and AC conceived and designed the database, supervised the data collection across updates, participated in data analysis and interpretation of results, was involved in the drafting of the manuscript and carried out critical revision for intellectual content. AC supervised database upkeep at the Centre for Mental Health Research, Australian National University until 2013, and KP currently supervises this at the Black Dog Institute, University of New South Wales. AC, BVS, JG, TD and KF, in addition to individuals mentioned below, undertook data collection and interpretation of results across various updates since 2009. Ongoing maintenance and refinement of the database will be collaboratively guided by the research team, and managed by HC. *Manuscript*: KP drafted the initial manuscript, and TD, KP, HC, AC, BVS, and JG undertook subsequent drafting and critical revisions of the manuscript. All authors read and approved the final manuscript.

## Pre-publication history

The pre-publication history for this paper can be accessed here:

http://www.biomedcentral.com/1471-244X/14/86/prepub
